# IntPred: a structure-based predictor of protein–protein interaction sites

**DOI:** 10.1093/bioinformatics/btx585

**Published:** 2017-09-18

**Authors:** Thomas C Northey, Anja Barešić, Andrew C R Martin

**Affiliations:** Institute of Structural and Molecular Biology, Division of Biosciences, University College London, London, UK

## Abstract

**Motivation:**

Protein–protein interactions are vital for protein function with the average protein having between three and ten interacting partners. Knowledge of precise protein–protein interfaces comes from crystal structures deposited in the Protein Data Bank (PDB), but only 50% of structures in the PDB are complexes. There is therefore a need to predict protein–protein interfaces *in silico* and various methods for this purpose. Here we explore the use of a predictor based on structural features and which exploits random forest machine learning, comparing its performance with a number of popular established methods.

**Results:**

On an independent test set of obligate and transient complexes, our IntPred predictor performs well (MCC = 0.370, ACC = 0.811, SPEC = 0.916, SENS = 0.411) and compares favourably with other methods. Overall, IntPred ranks second of six methods tested with SPPIDER having slightly better overall performance (MCC = 0.410, ACC = 0.759, SPEC = 0.783, SENS = 0.676), but considerably worse specificity than IntPred. As with SPPIDER, using an independent test set of obligate complexes enhanced performance (MCC = 0.381) while performance is somewhat reduced on a dataset of transient complexes (MCC = 0.303). The trade-off between sensitivity and specificity compared with SPPIDER suggests that the choice of the appropriate tool is application-dependent.

**Availability and implementation:**

IntPred is implemented in Perl and may be downloaded for local use or run via a web server at www.bioinf.org.uk/intpred/.

**Supplementary information:**

Supplementary data are available at *Bioinformatics* online.

## 1 Introduction

Protein–protein interactions are vital for the function of proteins, allowing them to carry out fundamental biological processes. Proteins interact via interfaces, areas of protein surface that are geometrically and physico-chemically complementary, allowing energetically favourable interactions to occur. Comparative analysis of human interaction databases shows that the number of complexes greatly exceeds the number of interacting proteins in humans (Futschik *et al.*, 2007) as well as in other species (Missiuro *et al.*, 2009). In yeast for example, the average number of interacting partners per protein has been estimated between 3 and 10 (Bork *et al.*, 2004). Typically, the more advanced the species is, the more connected the protein network is, indicating advancement in regulation of processes (Keskin *et al.*, 2008).

The main resource containing data on protein interfaces is X-ray crystallographic structures of protein complexes deposited in the Protein Data Bank (PDB). However, determining interfaces in this manner is costly and time-consuming. Furthermore, only 50% of structures in the PDB are protein complexes, the remainder being monomers or complexes with nucleotide chains, small peptides and ligand molecules. In addition, only a small fraction of true biological complexes—particularly transient complexes—is present in the PDB and verifying the presence of protein–protein interactions in a high-throughput manner is a hard problem. There is thus a need to predict interfaces *in silico*, to further the understanding of biological processes, as well as to inform drug design (Fletcher and Hamilton, 2006).

The nature of X-ray crystallography leads to crystal structures containing biologically irrelevant crystal contacts, or lacking relevant contacts. For biologically meaningful interfaces to be understood, biological contacts must be regenerated, or distinguished from crystal contacts. The ‘Protein, Interfaces, Structures and Assemblies’ (PISA) resource derives data from the PDB using a method based on chemical thermodynamics to distinguish macromolecular assemblies from non-biological crystal contacts (Krissinel and Henrick, 2007).

A large number of methods exist for the prediction of protein–protein interaction sites [for reviews, see de Vries and Bonvin (2008) and Esmaielbeiki *et al.* (2016)], the majority of which apply a machine learning method trained on a set of features derived from the sequences and/or structures of proteins with known interface sites. Prediction methods vary in the datasets used for training and testing, how interface residues are labelled, the nature of the interface type (i.e. transient and/or obligate), the nature of the prediction (e.g. patch- or residue-predictions), the selection of residues for evaluation (e.g. all or just surface residues), the features used and the machine learning method applied.

One of the biggest challenges in the field of protein–protein interface prediction is the lack of consensus on how methods should be evaluated and compared. In particular, benchmarking on independent test sets has shown that the performance of methods tends to be over-optimistically reported (Porollo and Meller, 2006; Zhou and Qin, 2007), which is most likely due to the common custom of reporting cross-validated performance on training data only, rather than testing on an independent dataset.

Some of the most commonly used features that have been shown to differ significantly between interface and non-interface residues include amino acid propensity scores (Lo Conte *et al.*, 1999), secondary structure (Neuvirth *et al.*, 2004), solvent accessibility (Jones and Thornton, 1997) and sequence conservation (Zhou and Shan, 2001). Generally these preferences have been exploited for prediction of protein–protein interfaces by using machine learning methods, including support vector machines (Bordner and Abagyan, 2005; Bradford and Westhead, 2005; Chung *et al.*, 2005; Koike and Takagi, 2004; Wang *et al.*, 2006) and neural networks (Chen and Zhou, 2005; Fariselli *et al.*, 2002; Ofran and Rost, 2003; Porollo and Meller, 2006). However, the random forest algorithm (Breiman, 2001) has been relatively underused for this purpose, despite its success in a range of biological problems, including activity prediction from chemical structure (Svetnik *et al.*, 2003), renal tumour classification (Shi *et al.*, 2005), detection of multiple-sclerosis-linked gene candidates (Goldstein *et al.*, 2010) and prediction of disease associated mutations (Al-Numair and Martin, 2013; Al-Numair *et al.*, 2016).

Here, the IntPred method for prediction of protein–protein interaction sites is presented. For a given PDB structure, IntPred uses sequence and structure information to create features that are the input to a random forest machine learning predictor, which will output a prediction label at either the surface patch- or residue-level. IntPred is cross-validated on a large set of structures obtained from PISA, as well as tested and compared with existing popular methods on an independent test set.

## 2 Materials and methods

### 2.1 Datasets

In order to create a training dataset, 58 397 biological units available in PISA were downloaded and both transient and obligate interfaces were included. Viral capsids and NMR entries were first removed, as were structures with resolution worse than 3 Å or R-factor greater than 30%. Peptide chains (<30 amino acids) were also removed and then any structure with more than one chain was kept, leaving 25 876 structures formed from 87 738 chains. To remove redundancy, these chains were clustered at 25% sequence similarity using PISCES (Wang and Dunbrack, 2003), culling ‘by chain’ and all other parameters set to their defaults. From each cluster, a representative chain was selected by choosing the chain with the best resolution or, if tied, the best R-factor. The final training set contained 4345 chains.

In order to create an independent test dataset, all the new biological units made available from the PISA resource over the following 5 months were obtained and filtered using the same procedure as described for the training set, with the exception that no clustering to remove redundancy was performed. This resulted in 4204 chains.

A dataset of obligate and transient interfaces was built from the independent test set using NOXclass (Zhu *et al.*, 2006), a high performance prediction method that predicts protein interactions as either obligate, non-obligate (transient) and/or crystal packing contacts. As NOXclass is run on a pair of interacting chains, a list was first created of all interacting chain pairs in the PQS files of the independent test set. NOXclass was run using all features except the ‘ConSurf score’ in multi-stage mode, where an interaction is first given percentage scores for the ‘biological’ and ‘crystal contacts’ labels and then another set of scores for the ‘obligate’ or ‘non-obligate’ labels (the ‘biological’ and ‘crystal contacts’ scores were ignored since PQS files should already have eliminated non-biological crystal contacts). Each pair was labelled ‘obligate’ if the ‘obligate’ score was higher than 50% and as ‘transient’ otherwise. Any PQS file that was predicted to contain both obligate and transient interfaces was discarded, leaving 916 obligate and 149 non-obligate PQS structures.

The content of the datasets is described in Supplementary file ‘Datasets.xls’.

### 2.2 Surface patch creation

In order to calculate the properties of subsets of a protein surface, it has to be divided into fragments. The program pdbmakepatch from the BiopTools tool set (Porter and Martin, 2015) was used to form overlapping surface patches from the protein surface.

Before introducing the algorithm implemented by pdbmakepatch, the following terms must be introduced:

- **Patch centre atom** is the central atom that is input to pdbmakepatch around which the patch is built. The residue to which the atom belongs is termed the ‘patch centre residue’.
- **Patch radius** is the threshold distance from the patch centre atom used to select candidate residues for inclusion within the final patch.
- **Contact radius** is defined for a pair of atoms as the sum of their van der Waals radii, plus a tolerance (here set to 0.2 Å). Two atoms are *in contact* if the distance between their centres is less than the contact radius.
- **Residue geometry vector** is a vector defined for a given residue with its initial point at the *C_α_* and its terminal point at the centre of geometry of the 10 spatially closest neighbours. The centre of geometry is calculated as the average of the neighbours’ *C_α_* coordinates.
- **Residue solvent vector** is also defined with its initial point at the *C_α_* of a given residue, but points in the opposite direction to the residue geometry vector.
- **Solvent angle** is defined between two residues and is the angle between the two residue solvent vectors.

For a given PDB file and a patch centre atom, pdbmakepatch iteratively builds a patch using the following procedure:

1. Define *P* as the initially empty set of atoms in the patch and add the patch centre atom to *P*.
2. Determine all residues with at least one atom centre within the patch radius from the patch centre atom. These are the set of residues *C* that are candidates for inclusion within the patch.
3. For each member of *P*, test if any of the members of *C* are in contact. If a member of *C* is in contact with a member of *P* and the solvent angle between them is less than 120° then move it to *P*.
4. Repeat step 3 until no more members of *C* are moved to *P*.
5. Label any residue with an atom in *P* as a patch residue.

**Fig. 1.**
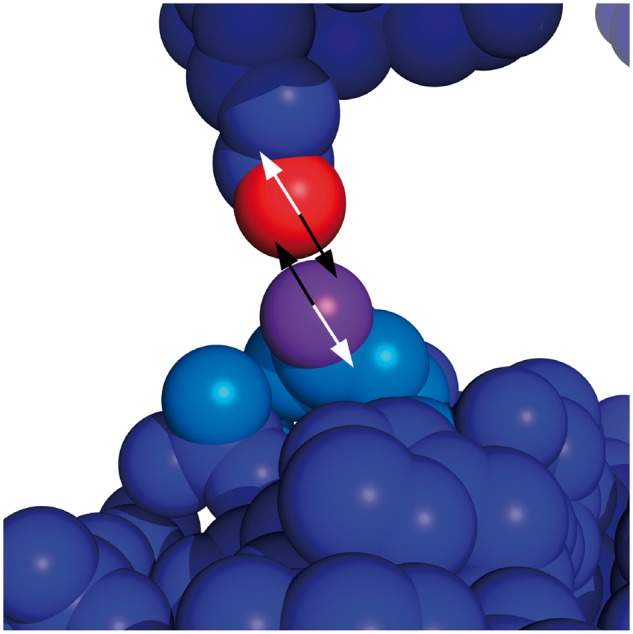
Residue geometry and solvent vectors. A candidate atom (red) is within the contact distance of a patch atom (purple). The residue geometry vectors (white) are used to calculate solvent vectors (black) and the angle between them is calculated. Because the angle is > 120°, the candidate atom is not included in the patch

The solvent angle test is used to avoid including residues from opposite sides of a pocket in the same patch, preventing the creation of discontinuous patches (see Fig. 1) (Jones and Thornton, 1997; Pettit *et al.*, 2007).

#### 2.2.1 Generating patches from a structure

For all of the structures used in this study, a set of overlapping patches was created to represent its surface. In order to create such a set, residues with relative solvent accessibility (*RASA*) >25% were identified. This is the set of patch centre residues. For each patch centre residue, the atom with the highest absolute solvent accessible area (*ASA*) is found. Each of these highly solvent accessible atoms is a patch centre atom that is input into pdbmakepatch.

Two different patch radii were tested: 9 and 14 Å. A 9 Å patch radius corresponds to the smallest biological interface found in the training set, whilst 14 Å corresponds to the minimum patch size needed for an interface to occur, according to Bogan and Thorn (1998).

#### 2.2.2 Assigning class labels

The class label of a patch is calculated by assessing the fraction of its total relative solvent accessible area (*RASA*) that is contributed by residues that have been defined as interface residues. A residue *i* is defined as interface if the following holds

(1) $$RASA_{i}^{n}-RASA_{i}^{c}\geq 10\%$$

where $RASA_{i}^{n}$ and $RASA_{i}^{c}$ are the non-complexed and complexed *RASA* values of *i* respectively. The ‘interface fraction’, $fASA_{p}$, for a patch *p* containing a set of residues *r_p_* and subset of interface residues $r_{intf}$ is calculated as

(2) $$fASA_{p}=\frac{\sum_{j\in r_{intf}}RASA_{j}^{n}}{\sum_{i\in r_{p}}RASA_{i}^{n}}$$

**Fig. 2.**
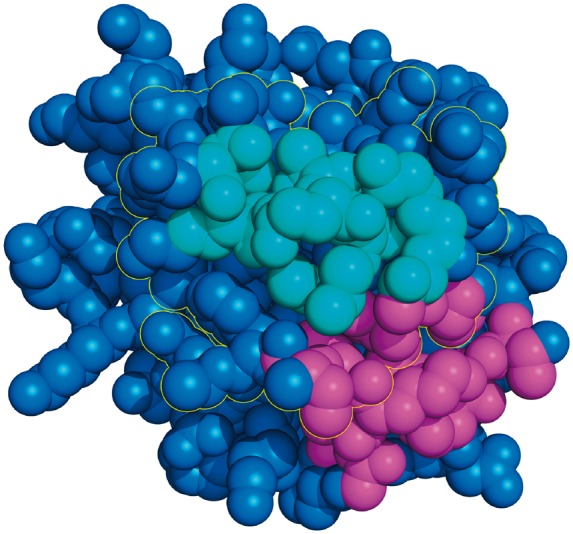
An example interface site (bordered in yellow), an interface patch (cyan) and a rim patch (magenta). The fraction of the rim patch’s surface involved in the interface is not high enough for the patch to be labelled as interface. See Equation 3

A class attribute value *C_p_* is then assigned for the patch as

(3) $$C_{p}=\{\begin{array}{cc} I, & \;\text{if}\;fASA_{p}\geq 0.5,\\ S, & \;\text{if}\;fASA_{p}=0,\\ U, & \text{otherwise}. \end{array}$$

where the value *U* corresponds to *unlabelled* and is assigned to patches that are on the rim of the interface (see Fig. 2). Patches with class attribute value *U* are excluded from training and testing at patch level to ensure that classification remains a binary problem, but are included during testing when patch predictions are mapped to residue predictions (see 2.6, ‘Mapping from patch to residue-level prediction’, below).

### 2.3 Features

IntPred uses 11 features for learning and prediction (summarized in Table 1) which can be divided into sequence features and structural features. The distributions of the residue-level features on which these patch-level features are based were all found to differ significantly between interface and non-interface (see Supplementary Figs S1–5).

**Table 1.** Summary of IntPred features

Feature	Description	Type
**Sequence**		
prop	propensity score	Continuous numeric
hpho	hydrophobicity	Continuous numeric
homology	homology conservation score	Continuous numeric
FEP	FEP conservation score	Continuous numeric
**Structural**		
SS	disulphide bonds	Continuous numeric
Hb	hydrogen bonds	Continuous numeric
helix (H)	*α*-helix secondary Structure	Binary categorical
sheet (E)	*β*-sheet secondary Structure	Binary categorical
mix (EH)	mixed secondary Structure	Binary categorical
coil (C)	coil secondary Structure	Binary categorical
pln	planarity	Continuous numeric
intf	Output class label	Binary categorical

*Note*: See text for description of how these features are calculated.

#### 2.3.1 Sequence features

The following features only take sequence-based properties into account. As these features are based on residue scores, the score of a patch is simply the average of the scores of its residues.

**Hydrophobicity:** The hydrophobicity of a residue is simply its hydrophobicity value on the Kyte and Doolittle hydrophobicity scale (Kyte and Doolittle, 1982).

**Propensity:** The propensity of a residue *i* of type *X* is calculated as

(4) $$Pr(i,X)=\left(ln\frac{F_{intf}(X)}{F_{surf}(X)}\right)\times \frac{ASA(i)}{\bar{ASA_{surf}}(X)}$$

where $F_{intf}(X)$ and $F_{surf}(X)$ are the interface and surface fractions (defined below) of residue type *X*, *ASA*(*i*) is the non-complexed absolute solvent-accessible area of residue *i* and $\bar{ASA_{surf}}(X)$ is the average absolute *ASA* for all surface residues of type *X*. The inclusion of *ASA*(*i*) means that the empirically obtained *ASA* of residue *i* is incorporated, rather than treating the contribution of every residue of type *X* as identical. Additionally, the inclusion of $\bar{ASA_{surf}}(X)$ controls for the difference in amino acid size, avoiding over-representation of bulky residues.

A positive propensity value indicates over-representation of residue type *X* in the interface set, while a negative propensity value indicates an under-representation.

For residue type *X*, the interface fraction $F_{intf}$ is calculated as

(5) $$F_{intf}(X)=\frac{\sum ASA_{intf}^{n}(X)}{\sum ASA_{intf}^{n}}$$

where the numerator is the total non-complexed absolute solvent accessibility for all training set interface residues of type *X* and the denominator is the total non-complexed absolute solvent accessibility of all interface residues.

Similarly, the surface fraction $F_{surf}(X)$ is calculated as

(6) $$F_{surf}(X)=\frac{\sum ASA_{surf}^{n}(X)}{\sum ASA_{surf}^{n}}$$

with corresponding values for the set of non-interface surface residues of the training set.

**Conservation scores**. For each residue, two conservation scores are calculated: a functionally equivalent protein (FEP) score and a homologue score. Each score is calculated on the basis of an alignment produced using the matches generated from two different resources.

In order to calculate FEP scores, PDBSWS (Martin, 2005) is used to determine an associated UniProtKB/SwissProt entry for a given PDB chain. The FOSTA resource (McMillan and Martin, 2008) is then used to find the family of functionally equivalent orthologues of which the entry is a member. If this family contains at least nine other members, then it is taken forward for alignment.

In order to calculate a homologue score, a BLAST search (Altschul *et al.*, 1990) against the UniProtKB/SwissProt database using the sequence of the PDB chain is undertaken, using default parameters. Matches containing any of the terms *putative*, *predicted* or *hypothetical* are discarded, as are matches with an E-value > 0.01. If a minimum of 10 sequence matches are retained, then up to 200 of the top hits (ranked by lowest E-value) are taken forward for alignment.

For each set of matches, Muscle Version 3.7 (Edgar, 2004) is used with default parameters to produce an alignment. Each alignment is used to calculate residue conservation scores using the ‘Valdar01’ method (Valdar and Thornton, 2001), implemented in our in-house program scorecons, part of the BiopTools package (Porter and Martin, 2015). For both conservation scores, the score of a patch is the average of the score of its residues.

#### 2.3.2 Structural features

The following features require structural information in order to be calculated.

**Averaged features:** Again, these features are calculated at the residue level and calculated for a patch by averaging the scores of its residues.

Intra-chain disulphide bonds are identified by using the pdblistss tool from BiopTools. pdblistss identifies disulphide bonds by searching for $S_{\gamma }$-pair distances of less than 2.25 Å. This distance measure is based upon the average disulphide $S_{\gamma }$ distance determined by Hazes and Dijkstra (1988), with an additional 10% tolerance for structure inaccuracy. A residue is given a score of 1 if it forms a disulphide bond or 0 otherwise.

Intra-chain hydrogen bonds are identified using the pdbhbond tool from BiopTools. pdbhbond identifies hydrogen bonds using the rules of Baker and Hubbard (1984). Given a donor atom *D* (to which the hydrogen is bound) and an acceptor atom *A*, where hydrogen positions can be calculated, a hydrogen bond is formed if the H⋯*A* distance is ≤2.5 Å and the angle at the hydrogen is 90–180°; where the hydrogen position cannot be calculated, the *D*⋯*A* distance must be ≤3.35 Å and the angle between the donor antecedent, *D* and *A* is 90–180°. A residue is given a score of 1 if it is involved in a hydrogen bond and 0 otherwise.

**Secondary structure:** Secondary structure is assigned to a residue using the pdbsecstr tool from BiopTools, which assigns secondary structure according to the method of Kabsch and Sander (1983). The secondary structure assignment of a patch *SS_p_* follows:

(7) $$SS_{p}=\{\begin{array}{cc} H & \;\text{if}\;\alpha \;>\;20\%\;\;\text{and}\;\beta \leq 20\%,\\ E & \;\text{if}\;\alpha \leq 20\%\;\;\text{and}\;\beta \;>\;20\%,\\ EH & \;\text{if}\;\alpha \;>\;20\%\;\;\text{and}\;\beta \;>\;20\%,\\ C & \;\text{if}\;\alpha \leq 20\%\;\;\text{and}\;\beta \leq 20\% \end{array}$$

where α and β are the percentages of residues assigned as α-helix and β-sheet respectively.

**Planarity:** Patch planarity is calculated by finding the root mean squared distance of all atoms of the patch from a plane of best fit. The plane of best fit is found by centring the (*x*, *y*, *z*) coordinates of the atoms of the patch and then undertaking PCA. The first and second primary components of the PCA define the plane of best fit.

### 2.4 Machine learning

All machine learning was performed using WEKA version 3.6.3 (Hall *et al.*, 2009; Witten *et al.*, 2011).

All supervised classifiers implemented in WEKA 3.6.3 were trained on the training dataset with a patch radius of 9 Å and evaluated using 10-fold cross-validation. It was found that no available machine learning method significantly outperformed the others (see Supplementary Fig. S6) and thus two models were carried forward for further testing: neural networks and random forests. Neural networks were chosen owing to their previous successful application in the field and random forests because of their success in other biological problems.

Neural networks were implemented using the ‘MultilayerPerceptron’ method in WEKA. Models with 5 hidden layer nodes (*H *=* *5) and 50 hidden layer nodes (*H *=* *50) were tested, but no improvement in performance was seen using *H *=* *50, while a 10-fold increase in the time to build the model was required.

The random forest algorithm implemented in WEKA was trained using 100 trees. 150 trees was also tested, but the improvements seen were too small to justify the increased time taken to train the forest. When there are *p* input features, it is recommended to use a feature bag size (*M_try_*) of $\sqrt{p}$ (Hastie *et al.*, 2009) and thus a feature bag size of 3 was chosen. Additionally, a range of feature bag sizes from 2 to 9 were tested but no improvement was seen.

Because *SS_p_* (equation 7) is a nominal value that has four possible values, WEKA converts it into four binary attributes. For both FEP and homologue scores, if insufficient sequences are available for the alignment to be performed, then missing values will be passed to the machine learning. WEKA deals with missing values for neural networks by imputing a value based on the mean of the distribution, while for random forests it uses the ‘fractional instances’ method. When a feature is used to split instances, any instances with missing features are sent to all child nodes, but weighted at each node according to the proportion of the number of instances at that node without a missing value and the total number of instances with no missing values across all child nodes.

The effect of using patch radii of 9 and 14 Å was tested, as well as different combinations of feature types.

### 2.5 Method performance measures

In order to evaluate the performance of a binary classifier, a number of different measurements can be used (see Supplementary Table S1). Overall, the Matthews’ Correlation Coefficient (MCC), which describes the correlation between the predicted and actual labels, is the most comprehensive measure since it is calculated using all four outcomes. However, MCC can hide an important trade-off between Sensitivity (the fraction of positive cases correctly labelled as positive) and Precision (the fraction of positively labelled cases that are actually positive, also known as the Positive Predictive Value) or Specificity (the fraction of negative cases correctly labelled as negative).

### 2.6 Mapping from patch to residue-level predictions

In order to compare the IntPred method with existing methods, residue-level predictions must be produced. This is done by mapping the prediction label of a patch to its central residue. Because only those residues that have an RASA>25% are defined as patch centre residues, those surface residues with an *RASA* between 10 and 20% will have no prediction label. Thus, in order to predict across all surfaces residues, these low-*RASA* surface residues are always predicted as non-interface.

### 2.7 Running existing methods

Interface predictions using IntPred were then performed using the independent test dataset described in the Materials and Methods. Several previously published protein–protein interface prediction tools were also assessed using this dataset: ProMate (Neuvirth *et al.*, 2004) was accessed through the web page (bioinfo.weizmann.ac.il/promate/) for batch queries using the default combination of scores and extracting amino acids coloured according to their probability of comprising an interface (set as the temperature factor in the PDB file). SPPIDER (Porollo and Meller, 2006) predictions were obtained from sppider.cchmc.org/, using the SPPIDER II classifier. PIER (Kufareva *et al.*, 2007) predictions were obtained from abagyan.ucsd.edu/PIER/pier.cgi as downloadable comma-separated value files. meta-PPISP (Qin and Zhou, 2007) and PINUP (Liang *et al.*, 2006) scores used within meta-PPISP were both obtained from pipe.scs.fsu.edu/meta-ppisp.html.

Each surveyed classifier provided residue-level predictions as numerical values. The same thresholds used in the original papers were used for all the methods to indicate a positive prediction (residue predicted as interface): *p* > 70 for ProMate; predicted by ≥5 neural networks for SPPIDER; score ≥30 for PIER; and *p* > 0.34 for meta-PPISP.

## 3 Results

### 3.1 Overall performance

Cross-validated performance was evaluated using different patch sizes and with different subsets of feature types. Table 2 shows the performance of the random forest which significantly out-performed the neural network (see Supplementary Table S2). In particular, a random forest trained on patches with a radius of 14 Å, with all features as input, performs best and this random forest model was titled ‘IntPred’ and carried forward for further testing.

**Table 2.** Random forest performance

	Attributes		Performance					
Patch radius	C _FEP_	C_HOM_	ACC	PREC	SPEC	SENS	MCC	F
SR	*✓*	*✓*	0.755	0.537	**0.944**	0.194	0.208	0.285
SR		*✓*	0.749	0.502	0.939	0.184	0.184	0.269
SR	*✓*		0.737	0.453	0.913	0.213	0.170	0.290
SR			0.710	0.370	0.875	0.218	0.114	0.274
9	*✓*	*✓*	0.760	0.679	0.906	0.439	0.398	0.533
9		*✓*	0.752	0.665	0.906	0.413	0.373	0.509
9	*✓*		0.750	0.651	0.894	0.433	0.374	0.520
9			0.733	0.608	0.881	0.405	0.327	0.486
14	*✓*	*✓*	**0.795**	**0.747**	0.894	**0.604**	**0.528**	**0.668**
14		*✓*	0.780	0.725	0.888	0.573	0.492	0.640
14	*✓*		0.780	0.718	0.882	0.582	0.492	0.643
14			0.764	0.691	0.871	0.555	0.453	0.616

*Note*: C_FEP_ =conservation score calculated over functionally equivalent proteins from FOSTA, C_HOM_ =conservation scores calculared from homologues collected by a BLAST search of UniProtKB/SwissProt. Structural attributes were used in all instances. SR, single-residue patches; ACC, accuracy; PREC, precision; SPEC, specificity; SENS, sensitivity; MCC, Matthews’ correlation coefficient; F, F-measure. The highest score in every column is shown in bold. *M_try_* (the number of randomly chosen attributes in every split) was set to 3 and *T* (the number of trees) was set to 100 in all cases, these having been found to provide the best performance (data not shown). All scores are averages over 10-folds of cross-validation.

The predictive performance of IntPred on the surface residues of the independent test set in comparison with existing methods is shown in Table 3. IntPred gives the highest precision of all methods, and thus one can be more confident that residues predicted as interface by IntPred are likely to be correct. Though SPPIDER has a lower precision and specificity than IntPred, its higher sensitivity leads to it having the highest MCC score of all the methods tested. However, SPPIDER also has the lowest specificity of all the methods tested. Thus, when comparing IntPred and SPPIDER, there is an obvious trade-off between sensitivity and precision/specificity: IntPred is more likely to miss a true interface residue than SPPIDER, but is more likely to be correct when it does predict a residue as interface. In contrast, SPPIDER over-predicts interface residues, leading to more true interface residues being correctly labelled, but also more non-interface residues being incorrectly labelled.

**Table 3.** Benchmarking of IntPred and other previously published general PPI methods using an independent test set

Method	ACC	PREC	SPEC	SENS	MCC	F
ProMate	0.780	0.401	**0.987**	0.031	0.058	0.057
PIER	0.754	0.511	0.932	0.214	0.207	0.302
SPPIDER	0.759	0.472	0.783	**0.676**	**0.410**	**0.556**
PINUP	0.772	0.459	0.927	0.220	0.199	0.298
meta-PPISP	0.755	0.499	0.902	0.300	0.245	0.375
IntPred	**0.811**	**0.564**	0.916	0.411	0.370	0.473
IntPred (patch)	0.771	0.803	0.922	0.522	0.500	0.633

*Note*: ACC, accuracy; PREC, precision; SPEC, specificity; SENS, sensitivity; MCC, Matthews’ correlation coefficient; F, F-measure. The highest score in every column is shown in bold. IntPred refers to the random forest model trained on all features and 14 Å-radius patches mapped to a residue-level prediction while IntPred (patch) refers to performance at the patch level.

Table 3 also shows the patch-level performance of IntPred on the independent test set. In comparison with residue-level prediction, patch-level performance is markedly better: specificity is similar, but precision is much higher. However, for patch-level predictions, only non-interface and interface patches were used to calculate evaluation statistics, ignoring predictions on *U*-labelled (rim) patches.

Examples of predictions for the light chain of mouse antibody HyHEL-5 (PDB code 1yqv chain L), *Bos taurus* actin-related protein 2/3 complex subunit 3 (PDB code 3dxk chain E), *Felis silvestris catus* hemoglobin-β chain (PDB code 3d4x, chain B) and a poorer prediction for *Salmonella typhimurium* uridine phosphorylase (PDB code 3dps, chain A) are shown in Supplementary Figures S7–10.

### 3.2 Obligate and transient complexes

The dataset used in training and evaluating IntPred was derived from the protein databank. Consequently it could be argued that many of these structures are obligate complexes, whose interface may be rather different from those in transient complexes (obligate complex interfaces tend to be more hydrophobic, dominated by aromatic residues, more conserved and larger). Indeed obligate interfaces are of less interest to a predictor that relies on structure since information on the interaction is already available in the crystal structure.

Consequently, a dataset derived from the independent test set, separated into obligate and transient complexes was evaluated using MCC with IntPred and the other five popular predictors (Table 4).

**Table 4.** Comparison of the performance of methods (assessed by MCC) on obligate and transient complexes

	MCC	
Method	Obligate complexes	Transient complexes
ProMate	0.037	0.166
PIER	0.288	0.217
SPPIDER	0.426	0.311
PINUP	0.205	0.235
meta-PPISP	0.257	0.268
IntPred	0.381	0.303

*Note*: Overall performance is show in Table 3.

IntPred does slightly better on obligate complexes than it did overall (MCC = 0.381 on obligate; MCC = 0.370 overall) and performs somewhat worse on transient complexes (MCC = 0.303). Notably, using MCC as an evaluator, IntPred maintains its second-ranked position on both obligate and transient complexes while SPPIDER again performs best. The performance of SPPIDER shows a similar trend to IntPred, being better on obligate complexes than overall (MCC = 0.426 on obligate; MCC = 0.410 overall) and somewhat worse on transient complexes (MCC = 0.311). Interestingly the drop in performance for SPPIDER on transient complexes is rather larger than that seen for IntPred closing the gap in their MCC performance.

In our evaluation, ProMate performs particularly badly overall, but has been trained specifically for use on transient complexes. As expected, its performance is even worse when tested only on obligate complexes, but increases by a factor of >2.8 when tested only on transient interfaces. Nonetheless, it remains the worst performing method in this evaluation.

## 4 Discussion

In this study, we have presented IntPred, a random forest machine learning predictor for the prediction of protein–protein interface sites. The method can predict at both the surface-patch level and the residue level. Testing of IntPred, as well as five popular methods, on an independent test set showed that IntPred outperformed all existing methods except SPPIDER, using MCC as a comparator. However, there is a sensitivity *vs.* precision/specificity trade-off between IntPred and SPPIDER such that one may be more suitable than the other given the problem in hand. If false positives are less tolerated than false negatives, then IntPred is preferable, whilst SPPIDER is more suitable for the converse. As with SPPIDER, IntPred performance assessed by MCC on a dataset of obligate complexes is slightly better than the overall performance, while on transient complexes it is somewhat worse. Nonetheless, the performance of IntPred on transient complexes is greater than the performance of all other methods (with the exception of SPPIDER) on obligate complexes or overall.

While the overall prediction performance is comparable with SPPIDER (trading sensitivity for precision/specificity), the comparison of random forests with neural networks (shown in Supplementary Table S2) illustrates the higher performance of random forests on this type of problem. Random forests are robust to over-prediction when non-orthogonal features (such as the two measures of conservation) are used as inputs.

Performance may be improved in the future by combining both IntPred and SPPIDER, along with other methods, in order to a produce a meta-predictor. The fact that the gap in MCC between IntPred and SPPIDER on transient complexes is much reduced suggests that, as the datasets increase in size, we should be able to train a version of IntPred solely on transient complexes and achieve better performance than SPPIDER. We also hope to exploit larger functional families (FunFams) developed by the Orengo group to improve the conservation score calculation (Das *et al.*, 2015).

The source code for IntPred is available at github.com/ACRMGroup/intpred/and IntPred is available to run via a web-server at www.bioinf.org.uk/intpred/.

## Supplementary Material

Supplementary Data

## References

[btx585-B1] Al-Numair N.S., Martin A.C. (2013) The SAAP pipeline and database: tools to analyze the impact and predict the pathogenicity of mutations. BMC Genomics, 14, S4. 10.1186/1471-2164-14-S3-S4 PMC3665582 23819919

[btx585-B2] Al-Numair N.S. et al (2016) The structural effects of mutations can aid in differential phenotype prediction of beta-myosin heavy chain (Myosin-7) missense variants. Bioinformatics, 32, 2947–2955. 27318203 10.1093/bioinformatics/btw362

[btx585-B3] Altschul S.F. et al (1990) Basic local alignment search tool. J. Mol. Biol., 215, 403–410. 2231712 10.1016/S0022-2836(05)80360-2

[btx585-B4] Baker E.N., Hubbard R.E. (1984) Hydrogen bonding in globular proteins. Prog. Biophys. Mol. Biol., 44, 97–179. 6385134 10.1016/0079-6107(84)90007-5

[btx585-B5] Bogan A.A., Thorn K.S. (1998) Anatomy of hot spots in protein interfaces. J. Mol. Biol., 280, 1–9. 9653027 10.1006/jmbi.1998.1843

[btx585-B6] Bordner A.J., Abagyan R. (2005) Statistical analysis and prediction of protein–protein interfaces. Proteins, 60, 353–366. 15906321 10.1002/prot.20433

[btx585-B7] Bork P. et al (2004) Protein interaction networks from yeast to human. Curr. Opin. Struct. Biol., 14, 292–299. 15193308 10.1016/j.sbi.2004.05.003

[btx585-B8] Bradford J.R., Westhead D.R. (2005) Improved prediction of protein–protein binding sites using a support vector machines approach. Bioinformatics, 21, 1487–1494. 15613384 10.1093/bioinformatics/bti242

[btx585-B9] Breiman L. (2001) Random forests. Mach. Learn., 45, 5–32.

[btx585-B10] Chen H., Zhou H.X. (2005) Prediction of interface residues in protein–protein complexes by a consensus neural network method: test against NMR data. Proteins, 61, 21–35. 16080151 10.1002/prot.20514

[btx585-B11] Chung J.L. et al (2005) Exploiting sequence and structure homologs to identify protein–protein binding sites. Proteins, 62, 630–640. 10.1002/prot.20741 16329107

[btx585-B12] Das S. et al (2015) Functional classification of CATH superfamilies: a domain-based approach for protein function annotation. Bioinformatics, 31, 3460–3467. 26139634 10.1093/bioinformatics/btv398 PMC4612221

[btx585-B13] de Vries S.J., Bonvin A.M. (2008) How proteins get in touch: interface prediction in the study of biomolecular complexes. Curr. Protein Pept. Sci., 9, 394–406. 18691126 10.2174/138920308785132712

[btx585-B14] Edgar R.C. (2004) MUSCLE: multiple sequence alignment with high accuracy and high throughput. Nucleic Acids Res., 32, 1792–1797. 15034147 10.1093/nar/gkh340 PMC390337

[btx585-B15] Esmaielbeiki R. et al (2016) Progress and challenges in predicting protein interfaces. Brief. Bioinf., 17, 117–131. 10.1093/bib/bbv027 PMC4719070 25971595

[btx585-B16] Fariselli P. et al (2002) Prediction of protein–protein interaction sites in heterocomplexes with neural networks. Eur. J. Biochem., 269, 1356–1361. 11874449 10.1046/j.1432-1033.2002.02767.x

[btx585-B17] Fletcher S., Hamilton A.D. (2006) Targeting protein–protein interactions by rational design: mimicry of protein surfaces. J. R. Soc. Interface, 3, 215–233. 16849232 10.1098/rsif.2006.0115 PMC1578744

[btx585-B18] Futschik M.E. et al (2007) Comparison of human protein–protein interaction maps. Bioinformatics, 23, 605–611. 17237052 10.1093/bioinformatics/btl683

[btx585-B19] Goldstein B.A. et al (2010) An application of Random Forests to a genome-wide association dataset: methodological considerations and new findings. BMC Genet., 11, 49. 20546594 10.1186/1471-2156-11-49 PMC2896336

[btx585-B20] Hall M. et al (2009) The weka data mining software: An update. SIGKDD Explor. Newsl., 11, 10–18.

[btx585-B21] Hastie T. et al (2009) The Elements of Statistical Learning, 2nd edn. Springer-Verlang, New York.

[btx585-B22] Hazes B., Dijkstra B.W. (1988) Model building of disulfide bonds in proteins with known three-dimensional structure. Protein Eng., 2, 119–125. 3244694 10.1093/protein/2.2.119

[btx585-B23] Jones S., Thornton J.M. (1997) Analysis of protein–protein interaction sites using surface patches. J. Mol. Biol., 272, 121–132. 9299342 10.1006/jmbi.1997.1234

[btx585-B24] Kabsch W., Sander C. (1983) Dictionary of protein secondary structure: Pattern recognition of hydrogen-bonded and geometrical features. Biopolymers, 22, 2577–2637. 6667333 10.1002/bip.360221211

[btx585-B25] Keskin O. et al (2008) Principles of protein–protein interactions: what are the preferred ways for proteins to interact? Chem. Rev., 108, 1225–1244. 18355092 10.1021/cr040409x

[btx585-B26] Koike A., Takagi T. (2004) Prediction of protein–protein interaction sites using support vector machines. Protein Eng. Des. Sel., 17, 165–173. 15047913 10.1093/protein/gzh020

[btx585-B27] Krissinel E., Henrick K. (2007) Inference of macromolecular assemblies from crystalline state. J. Mol. Biol., 372, 774–797. 17681537 10.1016/j.jmb.2007.05.022

[btx585-B28] Kufareva I. et al (2007) PIER: protein interface recognition for structural proteomics. Proteins Struct. Funct. Bioinf., 67, 400–417. 10.1002/prot.21233 17299750

[btx585-B29] Kyte J., Doolittle R.F. (1982) A simple method for displaying the hydropathic character of a protein. J. Mol. Biol., 157, 105–132. 7108955 10.1016/0022-2836(82)90515-0

[btx585-B30] Liang S. et al (2006) Protein binding site prediction using an empirical scoring function. Nucleic Acids Res., 34, 3698–3707. 16893954 10.1093/nar/gkl454 PMC1540721

[btx585-B31] Lo Conte L. et al (1999) The atomic structure of protein–protein recognition sites. J. Mol. Biol., 285, 2177–2198. 9925793 10.1006/jmbi.1998.2439

[btx585-B32] Martin A.C. (2005) Mapping PDB chains to UniProtKB entries. Bioinformatics, 21, 4297–4301. 16188924 10.1093/bioinformatics/bti694

[btx585-B33] McMillan L.E., Martin A.C. (2008) Automatically extracting functionally equivalent proteins from SwissProt. BMC Bioinformatics, 9, 418. 18838004 10.1186/1471-2105-9-418 PMC2576269

[btx585-B34] Missiuro P.V. et al (2009) Information flow analysis of interactome networks. PLoS Comput. Biol., 5, e1000350. 19503817 10.1371/journal.pcbi.1000350 PMC2685719

[btx585-B35] Neuvirth H. et al (2004) ProMate: a structure based prediction program to identify the location of protein–protein binding sites. J. Mol. Biol., 338, 181–199. 15050833 10.1016/j.jmb.2004.02.040

[btx585-B36] Ofran Y., Rost B. (2003) Predicted protein–protein interaction sites from local sequence information. FEBS Lett., 544, 236–239. 12782323 10.1016/s0014-5793(03)00456-3

[btx585-B37] Pettit F.K. et al (2007) HotPatch: a statistical approach to finding biologically relevant features on protein surfaces. J. Mol. Biol., 369, 863–879. 17451744 10.1016/j.jmb.2007.03.036 PMC2034327

[btx585-B38] Porollo A., Meller J.A. (2006) Prediction-based fingerprints of protein–protein interactions. Proteins Struct. Funct. Bioinf., 66, 630–645. 10.1002/prot.21248 17152079

[btx585-B39] Porter C.T., Martin A.C. (2015) BiopLib and BiopTools – a C programming library and toolset for manipulating protein structure. Bioinformatics, 31, 4017–4019. 26323716 10.1093/bioinformatics/btv482 PMC4673973

[btx585-B40] Qin S.B., Zhou H.-X. (2007) meta-PPISP: a meta web server for protein–protein interaction site prediction. Bioinformatics, 23, 3386–3387. 17895276 10.1093/bioinformatics/btm434

[btx585-B41] Shi T. et al (2005) Tumor classification by tissue microarray profiling: random forest clustering applied to renal cell carcinoma. Mod. Pathol., 18, 547–557. 15529185 10.1038/modpathol.3800322

[btx585-B42] Svetnik V. et al (2003) Random forest: a classification and regression tool for compound classification and QSAR modeling. J. Chem. Inf. Comput. Sci., 43, 1947–1958. 14632445 10.1021/ci034160g

[btx585-B43] Valdar W.S., Thornton J.M. (2001) Protein–protein interfaces: analysis of amino acid conservation in homodimers. Proteins, 42, 108–124. 11093265

[btx585-B44] Wang B. et al (2006) Predicting protein interaction sites from residue spatial sequence profile and evolution rate. FEBS Lett., 580, 380–384. 16376878 10.1016/j.febslet.2005.11.081

[btx585-B45] Wang G., Dunbrack R.L. (2003) PISCES: a protein sequence culling server. Bioinformatics, 19, 1589–1591. 12912846 10.1093/bioinformatics/btg224

[btx585-B46] Witten I.H. et al (2011). Data Mining: Practical Machine Learning Tools and Techniques, 3rd edn. Morgan Kaufmann, Burlington, USA.

[btx585-B47] Zhou H.X., Qin S. (2007) Interaction-site prediction for protein complexes: a critical assessment. Bioinformatics, 23, 2203–2209. 17586545 10.1093/bioinformatics/btm323

[btx585-B48] Zhou H.X., Shan Y. (2001) Prediction of protein interaction sites from sequence profile and residue neighbor list. Proteins, 44, 336–343. 11455607 10.1002/prot.1099

[btx585-B49] Zhu H. et al (2006) NOXclass: prediction of protein–protein interaction types. BMC Bioinformatics, 7, 27. 16423290 10.1186/1471-2105-7-27 PMC1386716

