# Supplementary material for: IntPred: a structure-based predictor of protein–protein interaction sites
Source: Bioinformatics. 2017 Sep 18;34(2):223–9. doi: 10.1093/bioinformatics/btx585 (PMC5860208; doi:10.1093/bioinformatics/btx585)
Supplement: Supplementary Data [file btx585_supp.zip › btx585-suppl_data/SupplementaryMaterial.pdf]

Supplementary Material

IntPred: a structure-based predictor of protein-protein interaction sites

Tom Northey, Anja Barešić and Andrew C.R. Martin

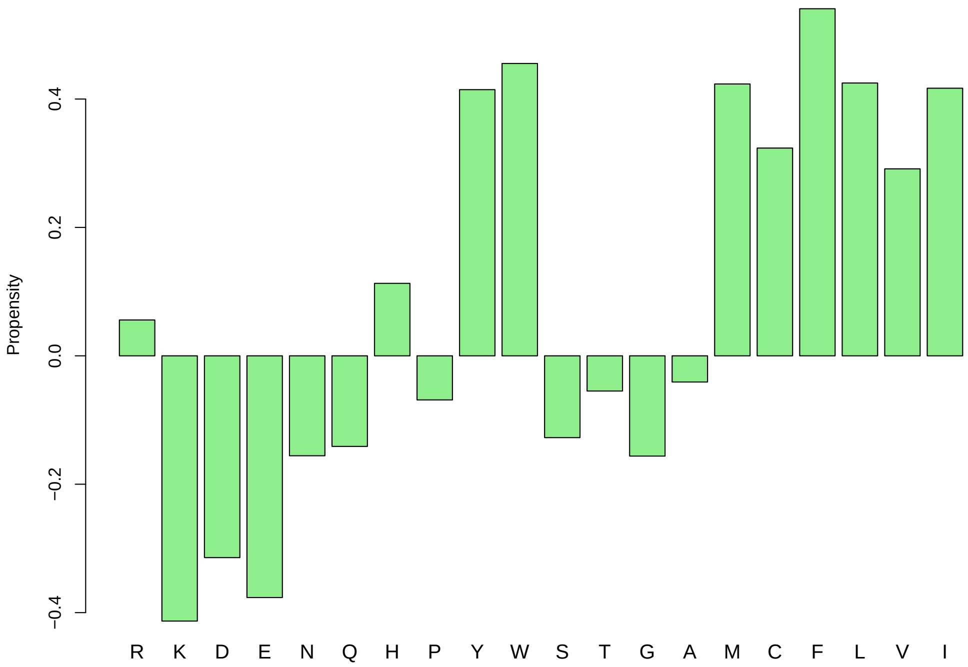

Supp Fig 1: Propensities of amino acid types in interface residues. Residue types are ordered by ascending hydrophobicity value according to the scale of Kyte and Doolittle (‘A simple method for displaying the hydropathic character of a protein’, *J Mol Biol*, **157**(1982),105–132)

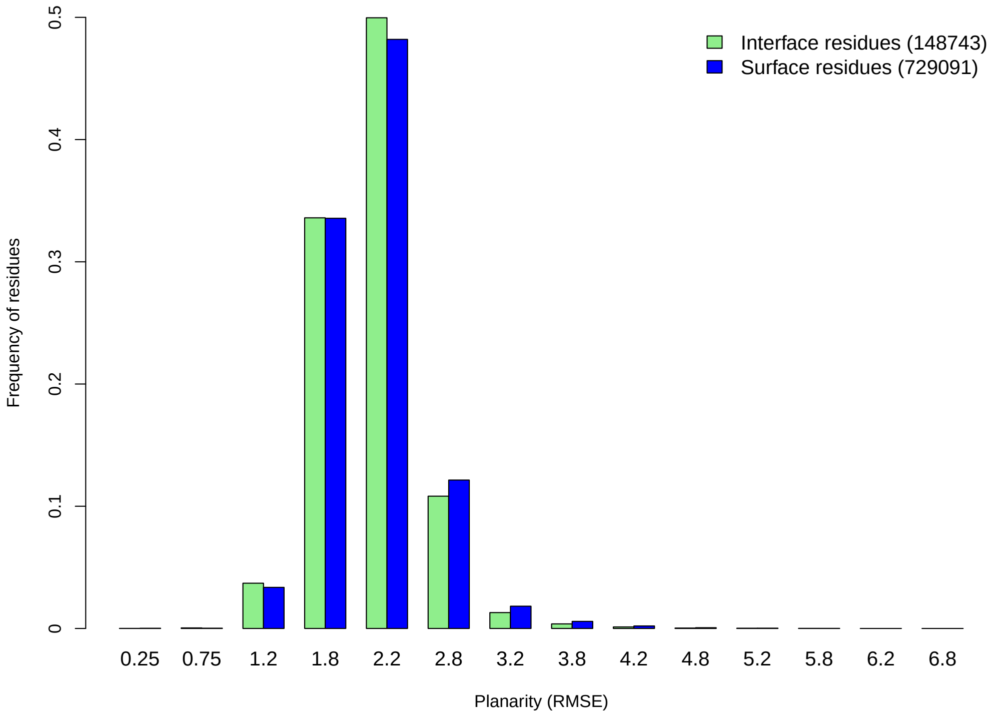

Supp Fig 2: Planarity values for interface and surface residues. There was a significant difference in planarity values between interface ($\bar{x} = 2.12$, $\sigma = 0.38$) and surface residues ($\bar{x} = 2.14$, $\sigma = 0.40$), $t(df = 223562.5) = -19.5522$, $p < 2.2 \times 10^{-16}$, when a two-tailed Welch two-sample t-test was performed. Total counts of residues per category are shown in the legend in brackets.

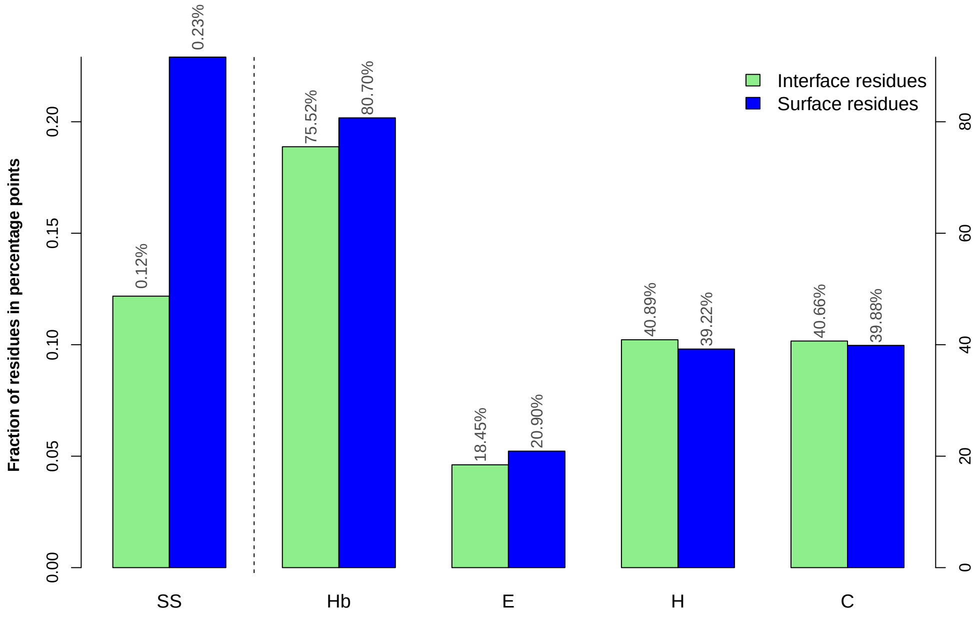

Supp Fig 3: Disulphide bonds, hydrogen bonds and secondary structure elements in interface and surface residues. Residue-level assignments of secondary structure elements are β, α or other, labelled with E, H and C, respectively. Frequency of disulphide bonds among residues is shown on a scale shown on the left, while the scale for hydrogen bonds and secondary structure fractions is shown on the right side of the graph. All categories show statistically significant difference between interface and surface categories (χ^2 test, $p < 0.01$), even after Bonferroni correction for multiple testing was applied.

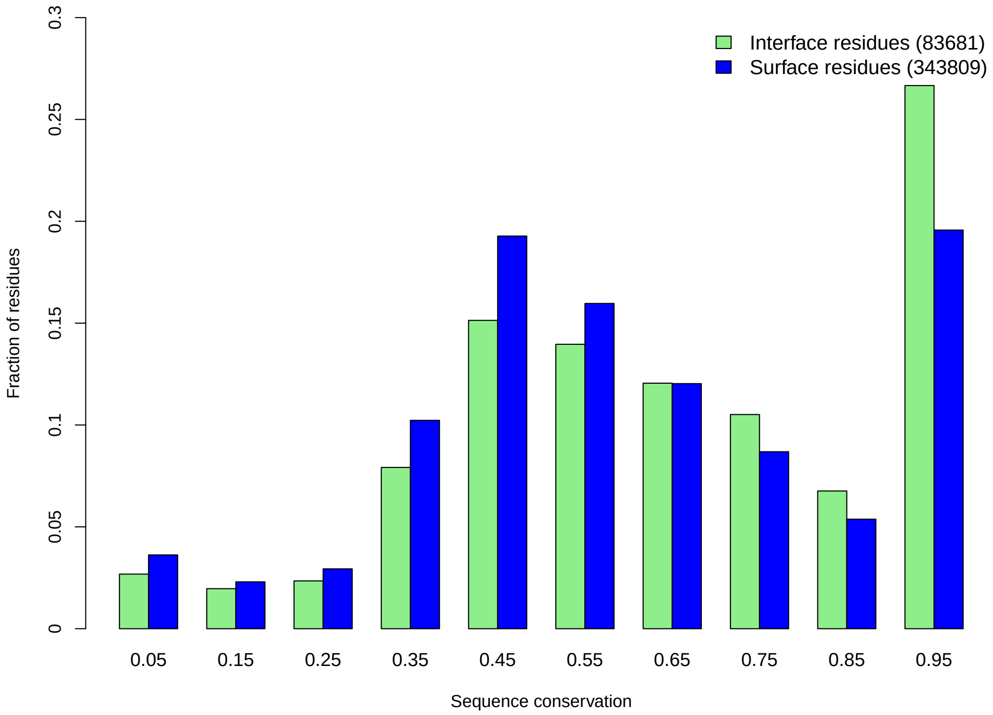

Supp Fig 4: FOSTA-based sequence conservation in interface and surface residues. There was a significant difference in rASA values between interface ($\bar{x} = 0.66$, $\sigma = 0.6$) and surface residues ($\bar{x} = 0.61$, $\sigma = 0.26$), $t(df = 38544.01) = 30.8154$, $p < 2.2 \times 10^{-16}$, when a two-tailed Welch two-sample t-test was performed.

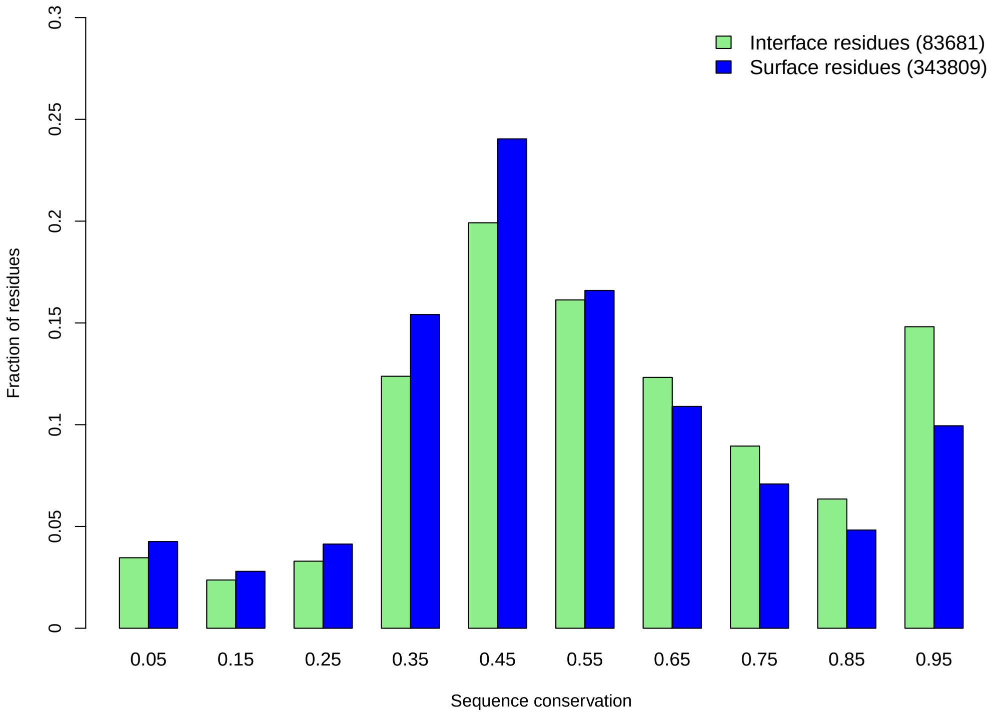

Supp Fig 5: BLAST-based sequence conservation in interface and surface residues. There was a significant difference in rASA values between interface ($\bar{x} = 0.58$, $\sigma = 0.25$) and surface residues ($\bar{x} = 0.53$, $\sigma = 0.23$), $t(df = 123168.8) = 52.7088$, $p < 2.2 \times 10^{-16}$, when a two-tailed Welch two-sample t-test was performed.

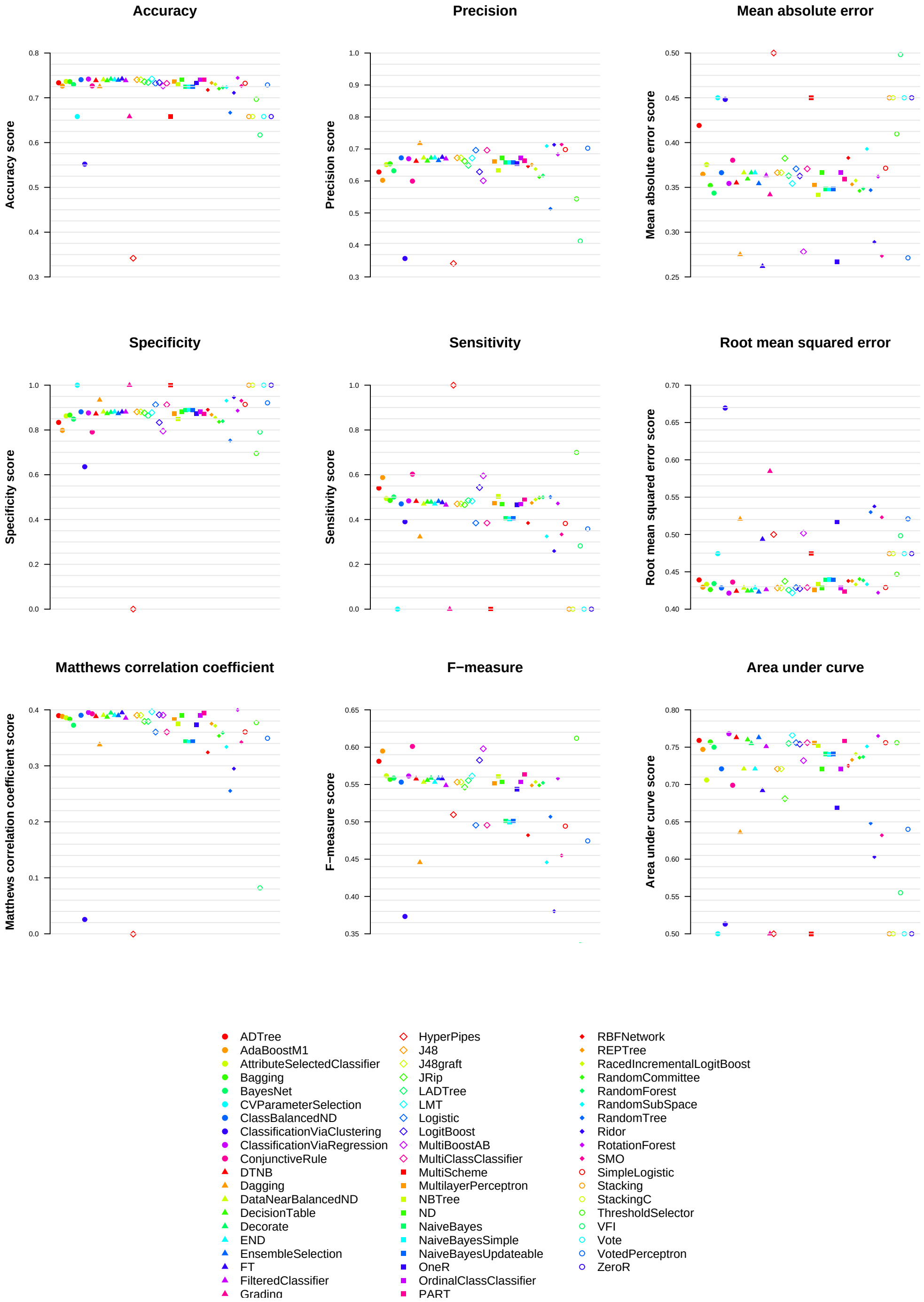

Supp Fig 6: A survey of machine learning tests used on interface data. Each score presented for a model is an average of 10 scores obtained during 10-fold cross-validation.

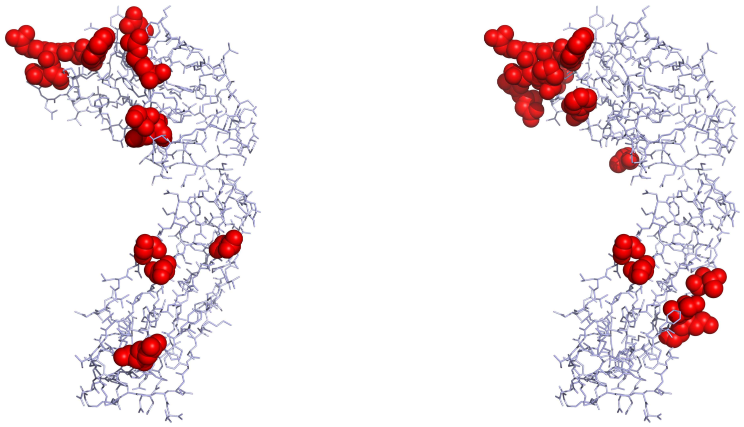

Supp Fig 7: The true interface (left) and predictions (right) for chain L of 1yqv (the light chain of *Mus musculus* antibody HyHEL-5).

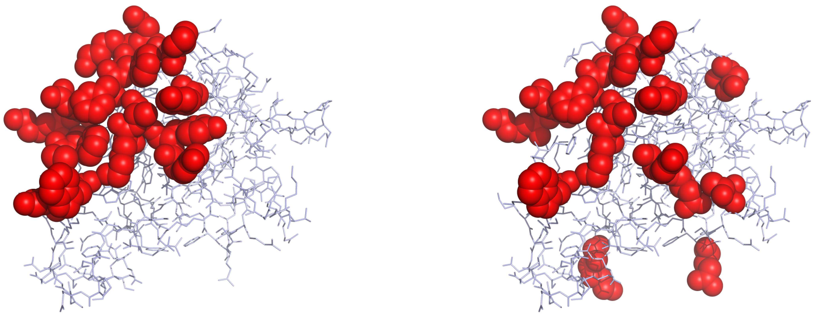

Supp Fig 8: The true interface (left) and predictions (right) for chain E of 3dxk (*Bos taurus* actin-related protein 2/3 complex subunit 3).

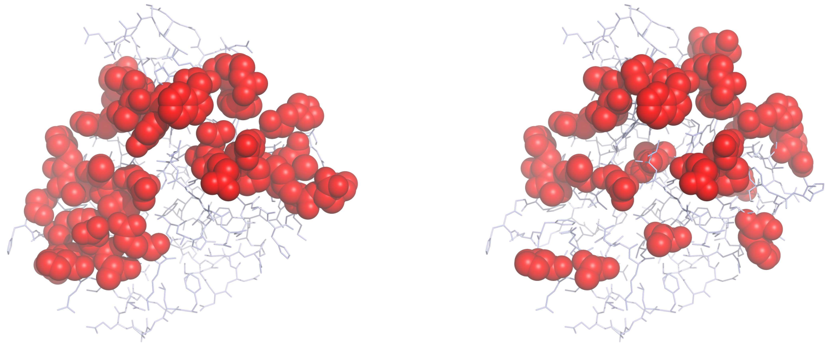

Supp Fig 9: The true interface (left) and predictions (right) for chain B of 3d4x (*Felis silvestris catus* hemoglobin-β chain).

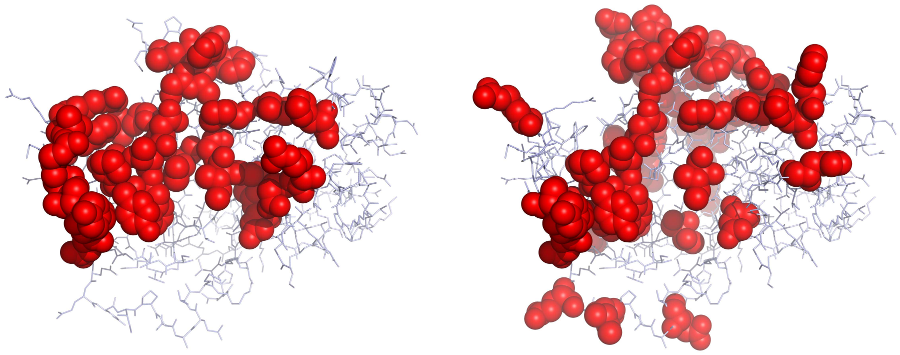

Supp Fig 10: The true interface (left) and predictions (right) for chain A of 3dps (*Salmonella typhimurium* uridine phosphorylase).

Measure	Formula	Range
Sensitivity	$\frac{TP}{TP+FN}$	$[0, 1]$
Specificity	$\frac{TN}{FP+TN}$	$[0, 1]$
Precision	$\frac{TP}{TP+FP}$	$[0, 1]$
Accuracy	$\frac{TP+TN}{TP+TN+FP+FN}$	$[0, 1]$
F1	$\frac{2TP}{2TP+FP+FN}$	$[0, 1]$
MCC	$\frac{TP \times TN - FP \times FN}{\sqrt{(TP+FP)(TP+FN)(TN+FP)(TN+FN)}}$	$[-1, 1]$

Supp Table 1: Binary classification performance measures. Sensitivity tells us the proportion of positive cases correctly labelled as positive and is the most important measure when the avoidance of false negatives is the primary concern. Specificity is the equivalent measure for negative cases and is useful when false positives are a concern. Precision is useful for understanding the likelihood of a positively-predicted instance being a true positive. Accuracy takes into account all prediction outcomes but can be misleading in the case of imbalanced data sets, where high accuracy can be obtained simply by labelling all instances with the majority class label. The F1 score is the harmonic mean of precision and sensitivity and as such is intended to give a single measure of how effective a classifier is. However, the F1 score does not take true negatives into account and therefore focuses only on the positive class. The better alternative is Matthews' Correlation Coefficient (MCC), which is calculated using all four outcomes. MCC is essentially the correlation between the predicted and actual labels and takes a value between -1 (perfect negative correlation) and 1 (perfect positive correlation) such that 0 means the classifier assignments are no better than random.

Patch	Attributes		Performance								
radius	C_{FEP}	C_{HOM}	ACC	PREC	SPEC	SENS	MCC	F	RMSE	MAE	AUC
SR	✓	✓	0.751	0.585	0.964	0.148	0.200	0.237	0.423	0.358	0.652
SR		✓	0.751	0.583	0.964	0.148	0.198	0.236	0.423	0.358	0.651
SR	✓		0.749	0.584	0.966	0.137	0.188	0.222	0.426	0.362	0.636
SR	✓*		0.760	0.597	**0.969**	0.138	0.198	0.225	0.417	0.348	0.661
SR			0.749	0.582	0.966	0.135	0.186	0.219	0.426	0.363	0.631
9	✓	✓	0.735	0.653	0.892	0.415	0.355	0.507	0.426	0.363	0.745
9		✓	0.736	0.653	0.892	0.417	0.356	0.509	0.426	0.363	0.745
9	✓		0.733	0.649	0.893	0.406	0.347	0.500	**0.429**	0.367	0.737
9	✓*		0.745	0.649	0.903	0.395	0.352	0.491	0.421	0.354	0.746
9			0.733	0.649	0.893	0.405	0.346	0.499	**0.429**	**0.368**	0.735
14	✓	✓	0.759	0.707	0.864	**0.574**	**0.462**	**0.634**	0.409	0.334	0.806
14		✓	0.759	**0.708**	0.865	**0.574**	**0.462**	**0.634**	0.409	0.334	0.806
14	✓		0.756	0.703	0.862	0.569	0.455	0.629	0.412	0.339	0.800
14	✓*		**0.766**	0.699	0.877	0.551	0.458	0.617	0.403	0.325	**0.808**
14			0.755	0.703	0.863	0.567	0.454	0.627	0.412	0.340	0.799

* instances with missing FOSTA value removed — 21% of the original dataset remained

Supp Table 2: Neural network performance.
C_{FEP}=conservation score calculated over functionally equivalent proteins from FOSTA, C_{HOM}=conservation scores calculared from homologues collected by a BLAST search of UniProtKB/SwissProt. Structural attributes were used in all instances. SR=single-residue patches, ACC=accuracy, PREC=precision, SPEC=specificity, SENS=sensitivity, MCC=Matthews' correlation coefficient, F=F-measure, RMSE=root mean squared error, MAE=mean absolute error, AUC=area under the curve. The highest score in every column is shown in bold. All scores are averages over 10-folds of cross-validation.
